# Application of Bionanomaterials in Tumor Immune Microenvironment Therapy

**DOI:** 10.1155/2021/6663035

**Published:** 2021-02-10

**Authors:** Jiawei Wang, Yan Bao, Yandan Yao

**Affiliations:** ^1^Breast Tumor Center, Sun Yat-sen Memorial Hospital, Sun Yat-sen University, Guangzhou 510120, China; ^2^Guangdong Provincial Key Laboratory of Malignant Tumor Epigenetics and Gene Regulation, Guangdong-Hong Kong Joint Laboratory for RNA Medicine, Sun Yat-sen Memorial Hospital, Sun Yat-sen University, Guangzhou 510120, China

## Abstract

Targeted therapy for the cancer immune system has become a clinical reality with remarkable success. Immune checkpoint blockade therapy and chimeric antigen receptor T-cell (CAR-T) immunotherapy are clinically effective in a variety of cancers. However, the clinical utility of immunotherapy in cancer is limited by severe off-target toxicity, long processing time, limited efficacy, and extremely high cost. Bionanomaterials combined with these therapies address these issues by enhancing immune regulation, integrating the synergistic effects of different molecules, and, most importantly, targeting and manipulating immune cells within the tumor. In this review, we will summarize the most current researches on bionanomaterials for targeted regulation of tumor-associated macrophages, myeloid-derived suppressor cells, dendritic cells, T lymphocyte cells, and cancer-associated fibroblasts and summarize the prospects and challenges of cell-targeted therapy and clinical translational potential in a tumor immune microenvironment in cancer treatment.

## 1. Introduction

The tumor immune microenvironment (TIME) is broadly populated with immune cells including tumor-associated macrophages (TAMs) and myeloid-derived suppressor cells (MDSCs) which suppress cancer immunity, leading to failure of immunotherapy [1]. Targeted therapy of the immune subset of TIME not only can efficiently remodel the TIME and activate the immune system against tumors but is also accompanied by adverse side effects mostly due to off-target toxicity [2]. For instance, the approved immune checkpoint blockade drugs which target cytotoxic T-lymphocyte antigen 4 (CTLA-4) and programmed cell death 1 (PD-1) or its ligand, programmed cell death ligand 1 (PD-L1), have shown efficacy in prolonging the overall survival of patients with various cancers. However, it also increases immune-related adverse events in patients, including the gastrointestinal tract and liver toxicity and endocrine dyscrasia [2, 3].

To enhance the curative effect and overcome the side effects of and traditional immunology therapy, developments in nanotechnology and bioengineering have provided a new approach that could greatly improve the safety and efficacy of cancer immunotherapy [4, 5]. Most clinical applications of bionanomaterials are as carriers of therapeutic and imaging agents in the treatment of cancer. Bionanomaterials have not only improved the delivery and efficacy of a series of pharmaceutical ingredients including drugs, antibodies, peptides, nucleotide, and enzymes but have also been designed to extend the duration of the release therapy and can be further modified to target specific sites in the body, thereby reducing the amount of the drug to achieve the desired therapeutic effect and reducing toxicity to the patients.

**Figure 1:**
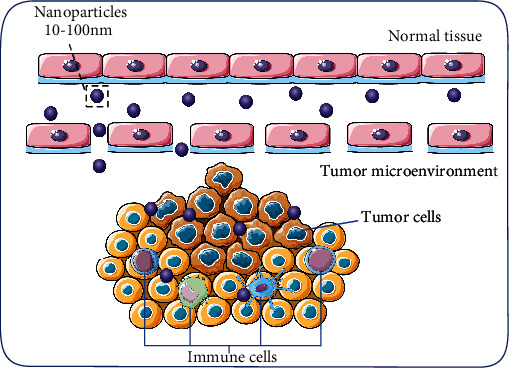
Overview of nanoparticles' permeation and retention effect (EPR). Nanoparticles ranging in size from 10 to 100 nm are aggregated by EPR effect through the immature blood vessels of the tumor and targeting the cells in the tumor microenvironment.

Bionanomaterials and related drug-delivery solutions focus the action of payloads on specific cell types and to specific anatomical locations to reduce adverse effects. Polymers and nanoparticles have become a focus of research in cancer therapy due to their potential ability to alter the pharmacokinetics and also accumulate in tumors through enhanced permeability and retention (EPR) effects [6, 7]. The EPR effect is that particles with sizes from 10 to 100 nm transport from the bloodstream, extravasate into tumors through the dysfunctional vasculature, and remain in tumors due to defective lymphatics, as shown in Figure 1. Besides, for the EPR effect, nanoparticle surface modification of specific markers is conducive to drug penetration into target immune cells. For example, the mannose receptor (CD206) has been demonstrated as an appealing target for M2 TAM in tumors and can be used in immunotherapy for TAMs and achieve good efficacy [8].

This review will summarize some of the recent advances in nanomaterial-based strategies of targeting immune cells in TIME, including TAMs, MDSCs, dendritic cells (DCs), T lymphocyte cells, and cancer-associated fibroblasts (CAFs). Instead of material design, we will focus on providing an overview of the material systems under development that is aimed at enhancing the effectiveness of cancer immunotherapy. Finally, we will summarize the prospects and challenges of targeting immune cells and clinical translational potential in TIME for cancer treatment.

## 2. The Mechanisms/Strategies for the Bionanomaterials in Regulating the Immune Cells

Many factors affect drug administration which may influence treatment outcomes, including pharmacokinetics, distribution, cellular uptake and metabolism, excretion and clearance, and toxicity [9]. In terms of this issue, bionanomaterials can be a good solution to treat cancer by delivering components to targeted immune cells and activate the immune system against tumors. Mostly, delivering small molecule immune activator/inhibitor or associated drug may be the most common way to regulate the immune cell in TIME, such as chemotherapy drug like Doxorubicin [10]. And recently, exosomes, nucleotides, antigens, and so on have been joined in the nanodelivery system [11–13]. Except for acting as the carriers, some nanoparticles (mostly mental nanoparticles) can regulate the immune cell directly. For instance, some nanoparticles could generate reactive oxygen species (ROS) and reprogram TAMs to an antitumor M1 phenotype directly, eradicating tumors effectively [14].

## 3. Targeted Therapy of Cells in the Tumor Immune Microenvironment

### 3.1. Tumor-Associated Macrophages (TAMs)

While immune infiltrates vary in different cancer types, monocyte/macrophages represent the major infiltrating population in most human cancers [15]. Recently, many studies have demonstrated their close relation with tumor progression and protumoral functions [16, 17], including tumor cell activation, angiogenesis, immunosuppression, tumor invasion, and metastasis [18]. Tumor-associated macrophages that differentiate from myeloid cells driven by the growing tumor signals are mostly classified as classically activated (M1) alternatively activated (M2) macrophages. Clinical immunohistochemical data has indicated that a higher density of total TAMs or M2 TAMs is associated with a poorer prognosis, while tumor infiltration with M1 TAMs may be a good prognostic factor in specific environments [16, 19–21]. Therapies targeted at TAMs are mostly divided into two strategies, including depletion and reprogramming [18]. However, the poor specific accumulation in tumors and significant toxicity of these agents have limited their use in the clinic.

Colony-stimulating factor 1 receptor (CSF-1R) is a canonical expressive marker of macrophages. Because macrophages are dependent on the CSF-1R signal, it is an attractive target for selectively depleting macrophages, and many associated small molecules targeting CSF-1R are under clinical trial and development [22–24]. For instance, platinum- (Pt-) prodrug conjugated small particles and BLZ-945, a small molecule inhibitor of CSF-1R, not only induce apoptosis of tumor cells but also modulate the tumor immune environment to eventually augment the antitumor effect of CD8+ cytotoxic T cells through TAM depletion [25]. Besides CSF-1R inhibitors, bisphosphonates are the drugs most commonly used to deplete TAMs in the clinic. To improve the overall effect and decrease toxicity, liposomal clodronate was created to promote TAM depletion and antitumor efficiency [26]. A more accurate way to specifically block the survival signal of M2 TAMs and deplete them from melanoma is to load anti-CSF-1R small interfering RNA (siRNA) on nanoparticles [11].

In addition to TAM depletion, reprogramming TAMs from M2 to M1 via nanomaterials have also been widely developed. Azide-modified exosomes derived from M1 macrophages, which are conjugated with antibodies of CD47 and SIRP*α*, actively target tumors, improve phagocytosis of macrophages, and reprogram macrophages from protumoral M2 to antitumoral M1 [12]. The trastuzumab-modified mannosylated liposomal system was able to repolarize the protumor M2 phenotype to the antitumor M1, reversing the resistance to tyrosine kinase inhibitor treatment in EGFR-mutated non-small-cell lung cancer [8]. Ferumoxytol, an iron oxide nanoparticle compound approved by the FDA for the treatment of iron deficiency, was found to have an intrinsic therapeutic effect on cancer growth due to macrophage polarization into proinflammatory M1 phenotypes [27]. Some nanomaterials can also directly stimulate the repolarization of TAMs. Nanoparticle-based reactive oxygen species photogeneration can reprogram TAMs to an antitumor M1 phenotype, effectively eradicating tumors [14].

It is worth noting that TAMs cannot be simply divided into two subtypes because TAMs may express both M1 and M2 markers [28, 29]. M1 and M2 may only represent two extreme examples of macrophage phenotype. Ratios of M1/M2 in tumor tissues and normal tissues might be a more suitable way to evaluate TAM phenotype-modulating nanomaterials.

### 3.2. Myeloid-Derived Suppressor Cells (MDSCs)

**Figure 2:**
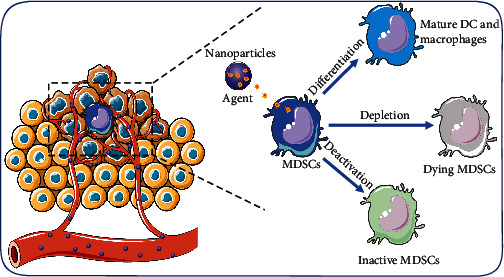
Nanomaterials for modulation of myeloid-derived suppressor cells (MDSCs). The immunotherapy strategy based on MDSC mainly includes the following three aspects: (i) induced to differentiate into mature DC and macrophages, (ii) depleted or blocked its amplification, and (iii) inhibited the immunosuppressive function.

Among cancer patients, there is an increase in immature myeloid cell proportion within TIME [30]. These cells, which express myeloid markers (Gr1+/CD11b+ in mice and CD11b+/CD33+ in human) [31], are named myeloid-derived suppressor cells because of their immunosuppressive and protumor characteristics. MDSCs can prevent immune cells from infiltrating tumors and also suppress effector T cell infiltration [32]. A meta-analysis has shown that a high level of MDSCs might be related to poor clinical outcomes of cancer patients; that is, MDSCs might be a potential biomarker in cancer treatment [33]. Similar to TAMs, various agents targeted at MDSCs have been developed, according to the strategies of depletion, differentiation, and deactivation, as shown in Figure 2.

Direct targeting and elimination of immunosuppressive MDSCs in TIME by signaling pathway regulation provide a new approach for tumor immunotherapy. PAH/RGX-104@PDM/PTX, a dual-pH-sensitive codelivery nanocarrier, not only causes apoptosis of cancer cells but also alleviates the immunosuppression of the TIME and finally enhances the antitumor effect of cytotoxic T lymphocytes (CTLs) through the depletion of MDSCs [34]. CpG-ODN/Poly(I:C)/RB6-8C5 nanoparticles have been developed which improve treatment outcome and have significantly reduced established B16 melanoma lung metastases through local depletion of MDSCs or reduction of immunosuppressive molecules (IFN-*α*, IL-10, Arg-1, and Nos2) which directly activate the natural killer (NK) cells and macrophages in the lung [35].

The polarization of immunosuppressive MDSCs in TIME of proinflammatory phenotype would be a better strategy than inhibiting or depleting it [36]. A designer scaffold encapsulated with Resiquimod (iNCV (R848)), which leads to the activation and maturation of antigen-presenting cells and induces the secretion of proinflammatory cytokines, can not only reduce the frequency of immunosuppressive cells in tumors but also increase systemic antitumor immune response while minimizing systemic toxicity [37]. Besides, zinc-doped iron oxide nanoparticles destroy glioma cells and repolarize MDSCs from an immunosuppressive phenotype to a proinflammatory phenotype *in vivo*, which promotes antitumor effects and synergistically promotes radiotherapy effects [38].

Some nanomaterials not only act as carriers for MDSC regulatory molecules but also directly silence or interfere with MDSCs. Low molecular weight heparin-tocopherol succinate nanoparticles prevent premetastatic niche formation by interfering with granulocytic myeloid-derived suppressor cells (G-MDSCs), effectively inhibiting implantation and colonization of circulating tumor cells [39].

Many nanomaterial agents have been developed for immunology cells as previously described; however, limited knowledge about the derivation and characteristics of myeloid-derived cells (TAMs and MDSCs) restricts accurate nanomaterial targeting.

### 3.3. Dendritic Cells (DCs)

DCs are a type of antigen-presenting cells (APC) that play an important role in the uptake and present tumor-associated antigens (TAAs) to the major histocompatibility complex (MHC), initiating antigen-specific T cell immune responses [40]. Cancer vaccines, composed of TAAs and adjuvants, act as the tumor antigen to stimulate DCs to generate TAA-specific CTL responses for killing tumor cells efficiently. Current cancer vaccines are designed to produce antibodies against cancer-causing viruses to reduce the risk of suffering from cancer. The best known is the human papillomavirus (HPV) vaccine, which can stimulate the body to produce antibodies to prevent HPV related to cervical, anal, oropharyngeal, vaginal, vulvar, and penile cancers. To date, three HPV vaccines (Gardasil, Cervarix, and Gardasil 9) have been approved by the United States Food and Drug Administration (FDA) [41]. Despite promising safety and immunogenicity profiles, the efficacy of the DC vaccine in clinical trials has not been satisfactory for several reasons, including method of loading, insufficient antigen presentation, poor accumulation in lymphatic tissues, and immunosuppression [42, 43]. Nanomaterials have obvious advantages in delivery capability and tissue targeting and have good application prospects in the development of tumor vaccines.

After intradermal injection, interstitial fluid flow transports ultrasmall nanoparticles highly efficiently into lymphatic capillaries and their draining lymph nodes (DLN), where dendritic cells were effectively activated [44]. Therefore, nanomaterial-based vaccines are capable of successfully transporting antigens to professional APCs in the DLN and enhancing immunogenicity [44, 45]. Silica nanoparticles as a lymph node targeting platform for vaccine delivery can accumulate in antigen-presenting cells in the draining lymph nodes after injection, greatly reducing the production of systemic proinflammatory cytokines and completely abrogating splenomegaly [13]. Besides receptor-mediated endocytosis, macropinocytosis is another way to take up exogenous antigens. For example, the biomimetic nanovaccine (R837-*α*OVA-ApoE3-HNP) can be taken into DCs through the macropinocytosis pathway and significantly promote DC maturation, antigen presentation, and strong T cell immune responses (including the generation of antigen-specific CD8+ T cells, expansion of IFN-*γ*+ CD8+ T cells, and the secretion of IFN-*γ*+) [46]. In addition, the nanoparticle can also be used as an adjuvant or immune enhancer and has the ability to activate cellular, humoral immunity and promote antigen presentation. For example, it has been proven that poly-l-lysine-coated nanoparticles were effective adjuvants and greatly enhance DNA immunogenicity [47].

### 3.4. T Lymphocyte Cells

Lymphocyte-mediated adaptive immune response plays an important role in the development of tumors and the dysfunction of the immune response in the TIME. CD8+ T cells differentiate to cytotoxic T cells, immigrate into the tumor microenvironment, and exhibit cytotoxicity and the ability to kill tumor cells. However, CD8+ T cells gradually produce a dysfunctional state known as T cell exhaustion after they infiltrate tumor tissues, characterized by losing robust effector functions and expressing multiple inhibitory receptors [48]. Mostly, the development of CD8+ T cell exhaustion could be due to persistent antigen exposure, inhibitory receptors, soluble mediators, and regulatory cells [48]. Substantially higher expressions of inhibitory receptors, including PD-1, CTLA-4, and T cell immunoglobulin, are the hallmarks of exhausted T cells. Immune checkpoint blockade therapy, which mostly targets PD-1/PD-L1 and CTLA-4/CD28 pathways in T cells to enhance antitumor immune responses, has led to important clinical advances and provided a new strategy against cancer [49]. However, multiple immune-related adverse events, including the gastrointestinal tract and liver toxicity, autoimmune disease, and endocrine dyscrasia, have been found in patients treated with immune checkpoint blockade, due to off-target effects [2]. Nanomaterial-engineered drug delivery systems and controlled release strategies can improve drug accumulation and retention within target cells and tissues and amplify their anticancer efficacy while reducing toxicities and off-target effects [50].

Controlled-release strategies for immune checkpoint blockade therapy may be an efficient way to enhance the antitumor effect. Spatiotemporally controlled nanodevices increase intratumoral drug concentrations and achieve sequential drug release, which enhances T cell infiltration in tumor tissues and thus prolongs the survival of mice [51]. Furthermore, a potent antitumor chemoimmunotherapy has been developed which utilizes tumor microenvironment-sensitive micelles bearing a sheddable PEG layer to mediate the site-specific sequential release of PD-1 monoclonal antibodies (MAbs) and Paclitaxel, resulting in a synergistic antitumor chemoimmunotherapy [52].

Materials not only enable controlled release of checkpoint blockade MAbs but can also be used to regulate the tumor microenvironment and promote checkpoint blockade MAb delivery and functions, such as increasing proinflammatory cytokine levels and T cell infiltration [53]. For example, photodynamic therapy (PDT) is able to stimulate antitumor immune responses by efficient photodynamic destruction of tumors to generate a mass of tumor-associated antigens and R837-containing nanoparticles as the adjuvants promote strong antitumor immune responses [54]. It has been shown that PDT with UCNP-Ce6-R837 in combination with CTLA-4 checkpoint blockade not only has excellent efficacy in eliminating tumors exposed to the near-infrared laser but also results in strong antitumor immunity to inhibit the growth of distant tumors left behind after PDT treatment [54].

Nanomaterial which delivers or loads cytokines acting on T cells provides another immunotherapy strategy. For example, transforming growth factor-*β* (TGF-*β*) and interleukin-2 (IL-2), which, respectively, suppress local tumor immune responses and amplify the activation of melanoma-specific T-cell responses, can be combined to treat metastatic melanoma [55]. Combination delivery of TGF-*β* inhibitor and IL-2 by nanoscale liposomal polymeric gels can deliver small hydrophobic molecular inhibitors and water-soluble protein cytokines in a sustained way to the tumor microenvironment to enhance tumor immunotherapy [55].

Besides immune checkpoint blockade therapy, chimeric antigen receptor T-cell (CAR-T) immunotherapy is another way to amplify cytotoxic T lymphocyte responses. CAR-T therapy, by genetically modifying and expanding T cells *ex vivo* before being infused back into patients, can concentrate tumor-specific CTLs in the tumor microenvironment. It has recently been approved by the FDA to treat large B-cell lymphoma and B-cell precursor acute lymphoblastic leukemia [56, 57] but is limited by low response rates, severe off-target side effects, cumbersome process, and extremely high cost [58]. For CAR-T therapy, biomaterials have been used to shorten the processing time, amplify the expansion of T cells *in vitro*, and promote the survival and proliferation of infused T cells [59]. For example, ionizable lipid nanoparticles (LNPs) are designed for *ex vivo* mRNA delivery to human T cells to induce functional protein expression, with substantially reduced cytotoxicity and potent cancer-killing activity [60]. The scarcity of tumor vessels and the immunosuppressive tumor microenvironment are often the reasons for the reduced efficacy of CAR-T cells in solid tumors. A combination of photothermal therapy with the adoptive transfer of CAR-T cells has superior antitumor activity in mice engrafted with human melanoma WM115 cell lines because it increases blood perfusion, releases antigens, and promotes the recruitment of endogenous immune cells [61].

### 3.5. Cancer-Associated Fibroblasts (CAFs)

The interaction between tumor cells and the surrounding stroma promotes the acquisition of an invasive phenotype, neoangiogenesis, progression, metastasis, immunosuppression, and chemoresistance of tumors [62]. Cancer-associated fibroblasts (CAFs) are the predominant cells in the tumor stroma (up to 80% in pancreatic cancer) [63] and exert an important influence on tumor growth by regulating the tumor microenvironment. It was found that the genes associated with colorectal cancer (CRC) recurrence and poor prognosis were upregulated mainly in CAFs, rather than in tumor cells [64]. Therefore, it has also been widely studied as a nanomaterial target for enhanced immunotherapy.

Recently, some of the biological properties of CAFs have been used to study and design new therapeutics and nanotherapeutics to modify TIME and improve the therapeutic activity of chemotherapy [65]. For example, due to the scarcity of tumor vessels and extensive deposits of extracellular matrix components, pancreatic ductal adenocarcinoma (PDA) may impute its unique chemoresistance to inefficient drug delivery [66]. PEGPH20 combined with IPI-926 specifically decreases the proliferation of stromal myofibroblasts, inhibits tumor growth, and prolongs survival when combined with gemcitabine in a genetically engineered mouse model of PDA. It does this by impeding the intratumoral vasculature of PDA and increasing the delivery of the chemotherapeutic drug [67]. Besides the combination therapy of nanomaterials and chemotherapy, nanoparticles affect the gene expression and secretion of CAFs, thereby altering their intrinsic interactions with malignant cells and affecting the protumor activity of the TIME. It has been demonstrated that Au-Ag nanoparticles achieve remarkable metastasis-suppressing activity by directly inhibiting adenocarcinoma cell proliferation, as well as indirectly by affecting cancer-associated fibroblasts by reducing their cancer-promoting function and regulating their secretory profiles [68]. In addition, due to the off-target distribution of anticancer nanoparticles to CAFs, researchers have exploited nanoparticles that can genetically modify CAFs into cells producing secretable TNF-related apoptosis-inducing ligand (sTRAIL) efficiently in situ, leading to apoptosis in the adjacent tumor cells in mice [69].

## 4. Future Outlook and Perspective

**Figure 3:**
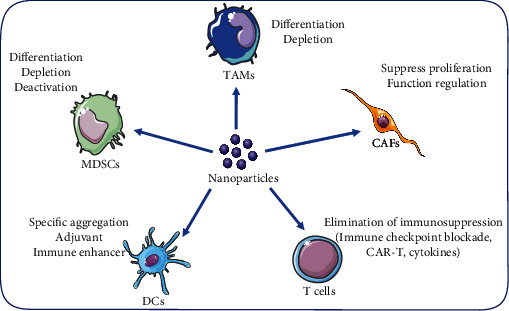
Some strategies of biomaterials in targeting different cells in a tumor immune microenvironment. Nanoparticles can target immune cells in TIME, including tumor-associated macrophages (TAMs), myeloid-derived suppressor cells (MDSCs), dendritic cells (DCs), T lymphocyte cells (T cells), and cancer-associated fibroblasts (CAFs), through various pathways or methods enumerated as shown.

For decades, cancer treatment has focused on killing tumor cells while ignoring other nontumor cells in the tumor microenvironment. In recent years, great attention has been paid to nontumor factors in the tumor microenvironment, as shown in Figure 3. Both CAR-T therapy and immune checkpoint blockade therapy, as well as treatments for other immune cells, have provided new solutions for cancer treatment which show efficacy for prolonging the overall survival of patients with various cancers. However, it also causes immune-related side-effects in patients and only benefits a fraction of patients.

Biomaterial carriers of immunotherapy can address the side effects of delivery and off-target effects, enhance immune regulation, integrate the synergistic effects of different molecules, and manipulate immune cells *in vivo* [70], as shown in Table 1. However, significant challenges remain to achieve a wide range of clinical outcomes for immune cell-targeted biomaterials. Firstly, the immune system has the paradoxical ability to have both tumor-suppressing and tumor-promoting roles, just as TAMs can be the proinflammatory M1 type and/or the anti-inflammatory M2 type. Furthermore, it is difficult to define MDSCs and TAMs through cell surface markers alone [28, 29]. There are some types of TIME cells and their main overexpressing receptors that could be targeted as shown in Table 2. The application of traditional fluorescence-based flow cytometry is limited by the number of phenotypic markers that can be detected. High-throughput approaches, such as mass spectrometry (CyTOF), that have emerged in recent years, should help further identify cell surface markers. Secondly, a tumor immune microenvironment is a complex system with mutual regulation among components, targeting only one kind of cells to antitumor seemed to be incomprehensive. Many other types of cells, particularly natural killer cells and B cells, may also provide effective targets for cancer immunotherapy [71] but have not been widely explored as targets for immunoregulatory materials. Finally, the shortcomings of nanomaterials themselves remain to be addressed, including damage to the cell directly or by initiating internal signaling pathways, the release of toxic material that impacts the organism's enzyme functions or cell DNA, and the generation of reactive oxygen species and subsequent oxidative stress [72].

Despite their potential advantages, only a handful of bionanomaterials have so far been used in clinical trials or received regulatory approval which affects the immune cell (Table 3). For now, a bionanomaterial drug like DOXIL, a kind of Doxorubicin-loaded liposomes, has been widely used in the clinic, which has the ability to kill the tumor directly meanwhile regulate T cells and myeloid cells [10]. With the recent outstanding achievements of the treatment of immune microenvironment cells and immune checkpoints, it is believed that nanomedicine materials will be widely used in clinical practice in the near future.

## Figures and Tables

**Table 1:** Examples of nanomaterial in modulation of TIME components.

TIME component	Strategy	Nanocarrier	Type	Active ingredient	Tumor model	Refs
TAMs	Depletion	Platinum -prodrug conjugated small particles	Pro-drug	BLZ-945	4T1, CT26	[25]
		Pegylated liposomal	Liposome	Clodronate	-	[26]
		SR-B1 linked with an M2 macrophage binding peptide	Peptide-lipid nanoparticle	Anti-CSF-1R siRNA	B16F10	[11]
	Reprogramming	Azide-modified exosomes	Exosome	Antibodies of CD47 and SIRP*α*	4T1	[12]
		Trastuzumab-modified, mannosylated liposomal	Liposome	Vorinostat	H1975	[8]
		ICG and titanium dioxide mannose-modified PEGylated PLGA nanoparticles	Inorganic nanoparticle	ICG	4T1	[14]
MDSCs	Depletion	Polymer of PEG-PDPA and PEG-PAEMA	Polymer	RGX-104	4T1	[34]
		CpG-ODN/Poly(I:C)	Polymer	Poly(I:C), RB6-8C5	B16	[35]
	Reprogramming	Porous scaffolds crosslinked with acid matrix	Polymer scaffold	Resiquimod (R848)	4T1, TC1	[37]
		Zinc-doped iron oxide nanoparticles	Iron oxide nanoparticle	Zinc	U87 MG, CT-2A	[38]
	Deactivation	LWMH -tocopherol succinate nanoparticle	Polymeric nanoparticle	D-*α*-tocopheryl succinate	B16	[39]
DCs	Lymph node targeting	Silica nanoparticle co-loading negatively charged oligonucleotide adjuvant and OVA antigen	Silica nanoparticle	Silica	EG.7-OVA	[13]
	Antigen presentation	R837-loaded 26 DMPC-PLGA hybrid nanoparticles	Polymer hybrid	R837, *α*OVA	B16-OVA	[46]
	Adjuvant or immune enhancer	Poly-l-lysine-coated nanoparticles load with sOVA-C1 plasmid	Polymer	OVA	EG7	[47]
T lymphocyte cells	Controlled release the drugs that target T cells	Nanoparticle with a micelle-liposome double-layer structure	Micelle/liposome	HY-19991	MCF-7	[51]
		Copolymer of azide-terminated polyethylene glycol and polyaspartic acid sheddable long-chain PEG	Polymer	PD-1 Abs	B16F10	[52]
	Enhanced delivery and function	Upconversion nanoparticles by co-loading chlorin e6, and imiquimod	Inorganic conversion	Imiquimod (R837)	CT26	[54]
	Through the action of cytokines	lipid bilayer surrounding a hydrogel core fabricated from a degradable polymer	liposomal polymeric	IL-2	B16-F10	[55]
	*In vitro* amplification of T cells	Ionizable lipid nanoparticles	lipid nanoparticle	mRNA	Nalm6	[60]
		PLGA nanoparticles loaded with ICG	Polymer	CSPG-4	WM115	[61]
CAFs	Target at stroma	PEGylated human recombinant PH20 hyaluronidase	Polymer	Hyaluronidase	KPC	[67]
	Deactivation	Gold-core silver-shell-structured hybrid nanoparticle system	Gold/polymer hybrid	Au, Ag	4T1	[68]
	Genetically modify	Lipid-coated protamine DNA complexes	Liposome	sTRAIL	BXPC3	[69]

Abbreviation: TAMs: tumor-associated macrophages, MDSCs: myeloid-derived suppressor cells, DCs: Dendritic cells, CAFs: Cancer-associated fibroblasts, SIRP*α*: signal regulatory protein*α*; SR-B1: scavenger receptor B type 1; PLGA: poly(lactic-co-glycolic) acid; ICG: indocyanine green; CpG-ODN/Poly(I: C): oligodeoxynucleotides containing CpG motifs/ polyinosinic-polycytidylic acid; LWMH: low molecular weight heparin; OVA: ovalbumin; CSPG4: antigen chondroitin sulfate proteoglycan-4; IL-2: interleukin-2; sTRAIL:secretable form of tumor necrosis factor related apoptosis-inducing ligand.

**Table 2:** Main overexpressing receptors that could be targeted in TIME.

Type of cells	Receptors
Tumor-associated macrophages	CD86, CD206, CD163, HLA-DR, CD11b, F4/80, MHC-II
Myeloid-derived suppressor cells	CD11b, CD14, CD15, CD66b, Ly6C, Ly6G
Dendritic cells	CD11c, MHC-II, CD1a
T lymphocyte cells	CD3, CD4, CD8, FOXP3, CD45RO, PD-1
Cancer-associated fibroblasts	GPR77, CD10, *α*SMA, FAP

**Table 3:** Some applications of bio-nanomaterial approved by FDA or in clinical trials in microenvironment therapy.

Name	Component	Immunotherapy effect	Clinical trial status	Ref
Dex	Nanoparticulate DC-derived exosomes	Stimulate CTLs and CD4+ T cells, NK cell activation	Phase 2, completed (NCT01159288)	[73]
Ferumoxytol	IONP	Transfer M2-like macrophages to M1-like in TME	Approved by FDA for anemia and kidney diseases	[27]
RNA-LPX (Lipoplexfi)	RNA-loaded liposomes	DC maturation, T cell response, inflammatory response	Phase 1, recruiting (NCT04503278)	[74]
DOXIL	Doxorubicin-loaded liposomes	Increased intratumoral CD8+ T cell infiltration, decreased the proportion of regulatory T cells (Treg cells), and increased CD80 expression by myeloid cells	Approved by FDA for cancer treatment	[10]
TRQ15-01	Nanogels	Ex vivo modification of T cells prior to adoptive-cell transfer	Phase 1, active (NCT03815682)	[75]
AST-008	CpG oligonucleotide nanoparticulate	Activating NK cells and inducing IFN-*α* production from plasmacytoid DC precursors, enhance B cell stimulatory property	Phase 1/2, recruiting (NCT03684785)	[76]

IONP: iron oxide nanoparticle; CTL: cytotoxic T lymphocyte; NK cell: natural killer cell; FDA: Food and Drug Administration; DC: dendritic cell.
